# Predominance and high antibiotic resistance of the emerging *Clostridium difficile* genotypes NAP_CR1_ and NAP9 in a Costa Rican hospital over a 2-year period without outbreaks

**DOI:** 10.1038/emi.2016.38

**Published:** 2016-05-11

**Authors:** Diana López-Ureña, Carlos Quesada-Gómez, Mónica Montoya-Ramírez, María del Mar Gamboa-Coronado, Teresita Somogyi, César Rodríguez, Evelyn Rodríguez-Cavallini

**Affiliations:** 1 Laboratory of Research in Anaerobic Bacteriology and Research Center in Tropical Diseases, University of Costa Rica, San José 11501-2060, Costa Rica

**Keywords:** antibiotic resistance, Costa Rica, emerging *Clostridium difficile*, NAP_CR1_, NAP9

## Abstract

*Clostridium difficile* is the major causative agent of nosocomial antibiotic-associated diarrhea. In a 2009 outbreak of *C. difficile*-associated diarrhea that was recorded in a major Costa Rican hospital, the hypervirulent NAP1 strain (45%) predominated together with a local genotype variant (NAP_CR1_, 31%). Both strains were fluoroquinolone-resistant and the NAP_CR1_ genotype, in addition, was resistant to clindamycin and rifampicin. We now report on the genotypes and antibiotic susceptibilities of 68 *C. difficile* isolates from a major Costa Rican hospital over a 2-year period without outbreaks. In contrast to our previous findings, no NAP1 strains were detected, and for the first time in a Costa Rican hospital, a significant fraction of the isolates were NAP9 strains (*n*=14, 21%). The local NAP_CR1_ genotype remained prevalent (*n*=18, 26%) and coexisted with 14 strains (21%) of classic hospital NAP types (NAP2, NAP4, and NAP6), eight new genotypes (12%), four environmental strains classified as NAP10 or NAP11 (6%), three strains without NAP designation (4%) and seven non-toxigenic strains (10%). All 68 strains were resistant to ciprofloxacin, 88% were resistant to clindamycin and 50% were resistant to moxifloxacin and rifampicin. Metronidazole and vancomycin susceptibilities were universal. The NAP_CR1_ and NAP9 strains, which have been associated with more severe clinical infections, were more resistant to antibiotics than the other strains. Altogether, our results confirm that the epidemiology of *C. difficile* infection is dynamic and that A^−^B^+^ strains from the NAP9 type are on the rise not only in the developed world. Moreover, our results reveal that the local NAP_CR1_ strains still circulate in the country without causing outbreaks but with equally high antibiotic-resistance rates and levels.

## Introduction

*Clostridium difficile* has become the leading cause of nosocomial diarrhea in adults.^1^ Clinical manifestations of *C. difficile* infections (CDI) vary from asymptomatic to fulminant colitis, including pseudomembranous colitis (PMC) or antibiotic-associated diarrhea. There may be complications, such as toxic megacolon, colonic perforation and a few extraintestinal manifestations.^2^

Most disease-causing isolates of *C. difficile* produce one or two toxins, i.e., TcdA and TcdB. These toxins enter intestinal epithelial cells and glycosylate various families of cytoplasmic GTPases,^3^ which leads to actin depolymerization with the loss of internal cell architecture, apoptosis, villus destruction and a mucosal inflammatory response.^4, 5^ The genes encoding toxins A and B (*tcdA* and *tcdB*) are part of a so-called pathogenicity locus (PaLoc), which also includes *tcdR* (a sigma factor that promotes the transcription of both of the toxin genes), *tcdE* (a potential holin) and *tcdC* (a potential negative regulator of *tcdA* and *tcdB*).^6^ Although deletions in *tcdC* have been claimed to favor TcdA and TcdB hypersecretion,^7^ this conjecture remains controversial because, as Cartman *et al.*^8^ demonstrated, these deletions have little effect in significantly increasing toxin production. Furthermore, variations in the combined repetitive oligopeptide domain of TcdB have been associated with increases in the virulence of epidemic strains.^9^ A minority of toxigenic *C. difficile* strains also produce a third toxin, known as binary toxin *Clostridium difficile* toxin (CDT), that is encoded by *cdtA* and *cdtB* at the CdtLoc locus and is separated from the PaLoc.^10^ CDT is composed of an ADP-ribosyltransferase that blocks actin polymerization and a binding component that is involved in toxin delivery.^11^ Although it is believed that CDT affects the cytoskeleton and enhances the adhesion and colonization of *C. difficile*,^10^ its role in CDI remains controversial.^12^

Various methods have been used to type *C. difficile* strains. While pulsed-field gel electrophoresis (PFGE) is predominantly used in North America, ribotyping by PCR is most often used in Europe. PFGE NAP1 strains correspond to ribotype 027 and harbor toxin A, toxin B, CDT, a 18-bp mutation in *tcdC* and a point mutation in this gene at position 117. In contrast, most TcdA-negative and TcdB-positive isolates belong to NAP9 and correspond to PCR ribotype 017.^13^

The effects, severity, complications, recurrence and even death rate of CDI have increased since 2003 in accordance with the increased isolation rates of hypervirulent strains, such as NAP1 and NAP9.^14, 15^ The NAP1 strains have been associated with higher sporulation rates and greater resistance to antimicrobials, especially fluoroquinolones,^14, 16^ whereas the NAP9 strains possess a TcdB that is capable of exerting a variant cytopathic effect.^17^

During a *C. difficile* outbreak in a major Costa Rican hospital in 2009, the hypervirulent NAP1 strain (45%) and the NAP_CR1_ strains (31%) were the predominant genotypes. Both types of strains were resistant to ciprofloxacin, moxifloxacin and levofloxacin, and the NAP_CR1_ strains were also resistant to clindamycin and rifampicin. NAP9 and the other seven classical nosocomial strains were present but in minor proportions.^18, 19^ Since this outbreak, the distribution of *C. difficile* genotypes in other Costa Rican hospitals has not been reported. To determine whether the NAP1 and NAP_CR1_ genotypes were dominant in a non-pediatric hospital over a two-year period without *C. difficile* outbreaks, 68 isolates from diarrheic patients were genotyped using PFGE and the antimicrobial susceptibilities of the isolates were tested. This information contributes to an understanding of CDI epidemiology worldwide and has the potential to guide local prevention efforts and treatment strategies.

## Materials and methods

### Isolates and bacteriological procedures

This study included 68 *C. difficile* isolates that were obtained from the diarrheal stools of non-pediatric patients who were admitted to a major hospital in Costa Rica with 633 beds between October 2010 and August 2012. All patients had been identified as having hospital-acquired CDI according to the criteria from the Infectious Diseases Society of America.^20^ Toxins A and B were detected in the stool samples by the hospital's clinical laboratory, and the samples with positive results were inoculated onto cefoxitin–cycloserine fructose agar plates (CCFA, Oxoid, Hampshire, UK). Yellow colonies on CCFA were cryopreserved at −80 °C in brain–heart infusion broth with 20% glycerol and sent to the Laboratory of Research in Anaerobic Bacteriology at the University of Costa Rica for further analysis and identification. There, the strains were subcultured in selective *C. difficile* moxalactam norfloxacin medium (Oxoid) and later on Brucella agar plates (BD Diagnostics, Franklin Lakes, NJ, USA) supplemented with 5% lysed horse blood (Oxoid) and 1 μg/mL vitamin K (Sigma-Aldrich, St. Louis, MO, USA) and (blood agar vitamin K) under an atmosphere composed of 90% N_2_, 5% H_2_ and 5% CO_2_ in an anaerobic chamber (Bactron II; ShellLab, Cornelius, OR, USA) at 37 °C for 48 h. The identities of the isolates were phenotypically confirmed using selective media, the rapID 32A system (bioMériuex, Marcy-l'Étoile, France) and chartreuse fluorescence on blood agar vitamin K under long-wave ultraviolet light and genotypically confirmed through PCR-based detection of the *C. difficile* marker *tpi* and molecular typing by PFGE.^21^

### Molecular typing

Genomic DNA from each strain was obtained from overnight cultures in brain–heart infusion broth (Oxoid) using the InstaGene reagent (Bio-Rad, Hercules, CA, USA). Fragments of *tcdA*, *tcdB*, *cdtB* and *tcdC* were amplified by PCR using known primers and conditions.^21^ A NAP1/027 strain (*tcdA*^+^, *tcdB*^+^, 18 bp-deletion in *tcdC*, *cdtB*^+^), a NAP7 strain (*tcdA*^+^, *tcdB*^+^, *tcdC* deletion>18 pb, *cdtB*^+^), an A^−^B^+^ strain (*tcdA^−^*, *tcdB*^+^, wild-type *tcdC*, *cdtB*^−^) and the non-toxigenic *C. difficile* strain ATCC 700057 (*tcdA^−^*, *tcdB*^−^, *tcdC*^−^, *cdtB*^−^) were used as controls.

For the PFGE typing, we obtained chromosomal *Sma*I macrorestriction patterns with a published method^19^ and a CHEF-DRIII variable angle system. Gel pictures were analyzed with BioNumerics v4.6 (Applied Maths, Austin, TX, USA) and compared with the databases of the National Microbiology Laboratory of Public Health Agency of Canada.

### Antibiotic susceptibility testing

The minimum inhibitory concentrations (MIC) for clindamycin, ciprofloxacin, moxifloxacin, rifampicin, metronidazole and vancomycin were determined using E-test strips (AB bioMérieux, Askim, Sweden) and Brucella agar plates containing 5% blood, 1 μg/mL vitamin K and 5 μg/mL hemin according to established guidelines.^22^ For susceptibility categorization, we used the resistance breakpoints recommended by the CLSI;^23^ i.e., 8 μg/mL for clindamycin, ciprofloxacin and moxifloxacin and 32 μg/mL for metronidazole. For rifampicin and vancomycin, we adopted the breakpoints recommended in the document M100-S21 for *Staphylococcus aureus* because no values have been defined for anaerobic bacteria; these values were 4 μg/mL for rifampicin and 16 μg/mL for vancomycin.

## Results

**Figure 1:**
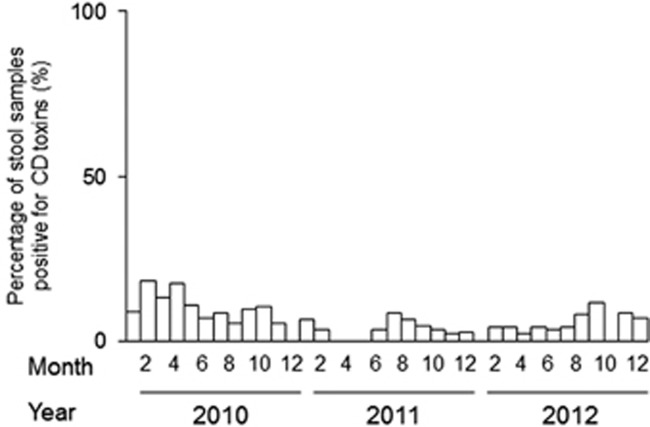
Epidemic curves for the stool samples that were positive for *C. difficile* (CD) toxins and were collected in a major hospital in Costa Rica from 2010 to 2012. The cases were diagnosed based on clinical evidence and toxin detection.

No outbreaks were reported from October 2010 to August 2012 in the hospital under study (Figure 1). Our genotyping procedure revealed that 28 isolates were positive for *tcdA* and *tcdB*, negative for *cdtB* and carried wild-type *tcdC;* these results were expected for classic hospital strains. Eighteen isolates exhibited the characteristic NAP_CR1_ pattern (i.e., *tcdA*^+^*, tcdB*^+^*, cdtB*^−^ and *tcdC* with a deletion), 14 exhibited the A^−^B^+^ strain pattern (i.e., *tcdA^−^, tcdB*^+^*, cdtB*^−^ and wild-type *tcdC*) and 1 isolate exhibited all 3 toxins and a deletion in *tcdC*. Seven isolates were non-toxigenic (Table 1).

**Figure 2:**
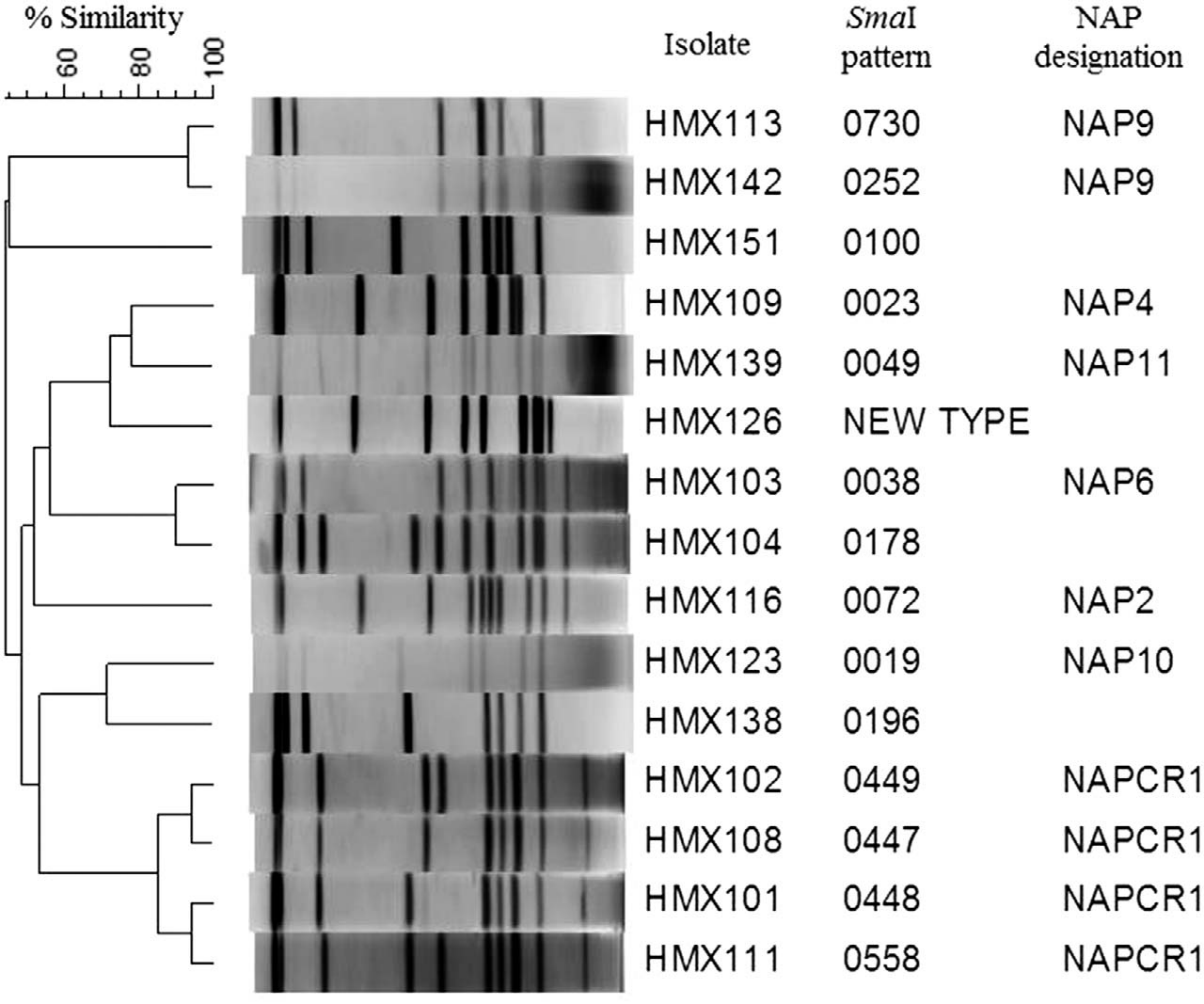
Pulse field gel electrophoresis results of representative *C. difficile* genotypes that were isolated between October 2010 and August 2012 from a hospital without a history of outbreaks during the period under study.

Although we observed a variety of genotypes (Figure 2 and Table 1), the local NAP_CR1_ genotype predominated (*n*=18, 26%). The NAP9 genotype was the second-most prevalent genotype (*n*=14, 21%), followed by the 14 isolates (21%) from the other traditional hospital pulsotypes of NAP2 (*n*=2), NAP4 (*n*=9), and NAP6 (*n*=3). Environment- or community-associated pulsotypes, such as NAP10 and NAP11, were observed (*n*=4, 6%) as were new pulsotypes (*n*=8, 12%) and known pulsotypes with no NAP designations (100, 196 and 178; *n*=3, 4%). Interestingly, no NAP1 strains were detected.

Antimicrobial susceptibility tests revealed that 88% of the isolates were resistant to clindamycin with very high MICs (>256 μg/mL, Table 2). Half of the isolates were resistant to moxifloxacin and rifampicin (MIC >32 μg/mL; Table 2). The MICs for metronidazole and vancomycin were rather low, and although all isolates were susceptible to both antibiotics, the MIC_90_ values were twice the MIC_50_ values (Table 2).

All isolates from the two most common genotypes (i.e., NAP_CR1_ and NAP9) were resistant to clindamycin, moxifloxacin and rifampicin (Table 1). Among all of the clindamycin-resistant strains, only those from the NAP_CR1_ and NAP9 genotypes exhibited MICs >256 μg/mL. The remaining hospital, community and non-toxigenic isolates and the strains from the new genotypes exhibited low antibiotic-resistance levels (Table 1).

## Discussion

We detected a predominance of *C. difficile* NAP_CR1_ and NAP9 strains in the diarrheal stool samples of patients admitted to a hospital in which no *C. difficile* outbreaks had occurred during the period under study. This knowledge is relevant from the clinical perspective because both genotypes have been associated with more severe cases of CDI.^19, 24, 25^

NAP_CR1_ strains have circulated in various Costa Rican hospitals since 2003 (López-Ureña D *et al.*, 2003, unpublished data) and have had major roles in the 2009 *C. difficile* outbreak in the San Juan de Dios Hospital.^18^ Here, we found NAP_CR1_ strains quite frequently in a group of clinical isolates from another hospital and confirmed the widespread distribution of these strains and their dominance even in the absence of outbreaks. Moreover, the identification of NAP_CR1_ PFGE types reveals the ongoing evolution of this lineage and this species over a short time.

The worldwide prevalence of clinically significant NAP9 strains seems to be increasing,^26^ particularly in Asian countries.^27^ Our results reinforce this view because this genotype was the second-most prevalent group. Many studies have found these strains in humans^24, 28^ and in animals.^29^ NAP9 strains have been found once in Costa Rica^18^ and in Latin America,^27^ where they seem to be gradually replacing other circulating genotypes. As observed in many countries,^30, 31^ our A^−^B^+^ strains were homogeneous, did not carry *cdtAB*, and harbored intact *tcdC* alleles. In contrast, in Australia, the *tcdA^−^, tcdB*^*+*^ strains are *cdtB*^*+*^.^13^ Furthermore, because our NAP9 strains were clindamycin-resistant, they may share a clonal origin with the strains that caused epidemics in Canada, the Netherlands, Ireland and Poland.^13^ We now know that these strains belong to the RT017 group (data not shown), but further studies are being performed to confirm that their sequence type is indeed ST37 or ST86.^32^

Although NAP1 strains were previously isolated during a *C. difficile* outbreak at another Costa Rican hospital, we only found a single *tcdA*^+^, *tcdB*^+^ and *cdtB*^+^ strain with a *tcdC* deletion in this study. This strain did not give rise to the 001 macrorestriction pattern associated with NAP1 in our previous reports^18, 19^ but rather exhibited a PFGE pattern without a NAP designation (i.e., a 0196 macrorestriction pattern). Other NAP strains coexisted including common inhabitants of hospital environments, such as NAP2, NAP4 and NAP6 strains,^33^ and NAP10 and NAP11 strains with potential zoonotic or community origins.^34^

Despite the marked increase in the recovery of clindamycin-resistant anaerobic strains in Costa Rica in the last decade,^35, 36^ this antibiotic is still the first-choice antibiotic for infections by anaerobic bacteria in Costa Rica and other geographic areas.^37^ The alarming clindamycin resistance level of *C. difficile* observed in this study (88%) is slightly lower than the level recorded during a 2009 outbreak at another major hospital (97%)^18^ but is still much higher than the values reported from other latitudes.^38, 39^ Almost half of the strains, all of which belonged to genotypes NAP_CR1_ and NAP9, had MICs >256 μg/mL, whereas the remaining strains had MICs=8 μg/mL. These findings indicate that only certain lineages acquire highly efficient mechanisms of resistance to clindamycin.

Fluoroquinolone resistance is increasing in epidemic strains of *C. difficile* primarily due to the emergence of chromosomal mutations in DNA gyrase genes.^38, 39, 40^ As described in 2010, all strains from this study were resistant to ciprofloxacin with MICs ⩾32 μg/mL.^18^ Moreover, half of the isolates were resistant to moxifloxacin. All of the NAP_CR1_ and NAP9 strains were resistant to both quinolones. Older fluoroquinolones, such as ciprofloxacin, exhibited moderate or poor activity against *C. difficile*, and the third- and fourth-generation fluoroquinolones, such as moxifloxacin, were effective against these bacteria. However, recent studies indicate that the rates of resistance to moxifloxacin in *C. difficile* have increased dramatically in different countries.^41, 42, 43^

Resistance to rifampicin is not unusual in *C. difficile* (6%–39%), especially in multidrug-resistant strains.^44, 45^ The reported MIC values of ⩽0.002 μg/mL for susceptible strains and >32 μg/mL for resistant strains largely match our findings.^44, 46^ Up to 50% of our strains were rifampicin-resistant, particularly those from the NAP_CR1_ and NAP9 genotypes, which were also resistant to clindamycin, ciprofloxacin and moxifloxacin. Interestingly, all of the isolates that were resistant to moxifloxacin were also rifampicin-resistant, and this association should be explored further.

The antibiotic-resistance levels of the less abundant genotypes were rather low, but they supported the previously reported increased resistance to fluoroquinolone of non-epidemic *C. difficile* strains^40^ and the ciprofloxacin resistance that is present in virtually all strains of *C. difficile*.

All of the isolates were susceptible to metronidazole and vancomycin. In agreement with the known metronidazole MIC of *C. difficile*,^20^ our MICs were invariably <2.0 μg/mL. However, a gradual change in the pattern of sensitivity to this antibiotic might be occurring within our strains because the MIC_90_ for metronidazole was twice the MIC_50_. A similar situation might be occurring in terms of their vancomycin susceptibility because 12 strains (18%), six of which were classified as NAP_CR1_, exhibited reduced vancomycin susceptibility with MICs ⩾2 μg/mL. These data strongly support the presence of continuous monitoring programs both in clinics and the community.

In this study, a local genotype of *C. difficile* was the most prevalent strain in a set of 68 isolates that were recovered in a hospital over a 2-year period without outbreaks. This genotype has previously been found to be dominant in another hospital during an outbreak. The second-most prevalent genotype was NAP9, which is in line with its increased prevalence in the USA,^31^ Europe,^24, 31^ Asia^25^ and Australia.^13^ The NAP_CR1_ and NAP9 genotypes exhibited high levels of antibiotic resistance, which reflects the use of antibiotics to which the strains have developed resistance and the association between CDIs and increased antibiotic use. The high levels of resistance to several antibiotics among the predominant genotypes could favor their persistence in the hospital environments and dominance over other genotypes. Constant characterization of circulating *C. difficile* isolates in terms of their population structures and antibiotic resistances not only improves our understanding of the epidemiology of CDI but also guides sanitary authorities and physicians in efforts to reduce the burden associated with this emerging pathogen.

## Figures and Tables

**Table 1:** Antibiotic resistances of various *Clostridium difficile* genotypes recovered between October 2010 and August 2012 at a hospital without a history of outbreaks during the period under study

		**Resistance (% isolates)**					
**Genotype**	**Number of isolates(%)**	**Clindamycin**	**Ciprofloxacin**	**Moxifloxacin**	**Rifampicin**	**Metronidazole**	**Vancomycin**
NAP_CR1_/012	18 (26%)	100	100	100	100	0	0
NAP9/017	14 (21%)	100	100	100	100	0	0
NAP2, NAP4, NAP6	14 (21%)	93	100	7	0	0	0
NAP10, NAP11	4 (6%)	75	100	0	0	0	0
New genotypes	8 (12%)	88	100	12	8	0	0
No NAP designation^a^	3 (4%)	25	100	25	25	0	0
Non-toxigenic	7 (10%)	71	100	0	0	0	0

a Includes *Sma*I patterns 100, 196 and 178.

**Table 2:** MICs and resistances of 68 isolates of *Clostridium difficile* recovered between October 2010 and August 2012 from a Costa Rican hospital without a history of outbreaks during the period under study

**Antibiotic**	**MIC range (μg/mL)**	**MIC_50_ (μg/mL)**	**MIC_90_ (μg/mL)**	**Resistance (% isolates)**
Clindamycin	1.5–>256	256	256	88
Ciprofloxacin	>32	>32	>32	100
Moxifloxacin	0.75–>32	>32	>32	50
Rifampicin	<0.002–>32	>32	>32	50
Metronidazole	0.064–1.5	0.4	1	0
Vancomycin	0.38–4.0	1	2	0

Abbreviation: minimum inhibitory concentration, MIC.
